# Effectiveness of a Dyadic Technology–Enhanced Home-Based Horticultural Therapy on Psychosocial Well-Being Among People With Dementia and Their Family Caregivers: Multimethods Pilot Study

**DOI:** 10.2196/66017

**Published:** 2025-02-05

**Authors:** Patrick Pui Kin Kor, Justina Yat Wa Liu, Arkers Kwan Ching Wong, Alex Pak Lik Tsang, Han Zhi Tan, Daphne Sze Ki Cheung, Humphrey Kwong Wai Leung, Frances Kam Yuet Wong

**Affiliations:** 1 School of Nursing The Hong Kong Polytechnic University Hong Kong China (Hong Kong); 2 Research Institute for Smart Ageing PolyU Academy for Interdisciplinary Research The Hong Kong Polytechnic University Hong Kong China (Hong Kong); 3 School of Nursing and Midwifery, Faculty of Health Deakin University Melbourne Australia; 4 Centre for Quality and Patient Safety Research/Alfred Health Partnership Institute for Health Transformation Deakin University Melbourne Australia; 5 University Fellows Association The Hong Kong Polytechnic University Hong Kong China (Hong Kong)

**Keywords:** horticultural activity, dementia, caregivers, dyadic intervention, technology–enhanced intervention

## Abstract

**Background:**

Horticultural therapy (HT) has been proposed to be an effective intervention for improving the psychosocial well-being of people with dementia and their caregivers. However, constraints such as limited land space in high-density cities, unstable weather, and lack of gardening experience may hamper the delivery of HT to people with dementia and their caregivers.

**Objective:**

This pilot study aimed to examine the feasibility and preliminary effects of a technology-enhanced home-based HT for people with dementia and their caregivers using a hydroponic indoor growing system.

**Methods:**

A single-group pre-post design was adopted. A total of 37 dyads of people with dementia and their caregivers participated in 3 weekly face-to-face sessions, followed by 8 weeks of home-based horticultural activities. Outcomes were measured at baseline and postintervention (at week 11), including feasibility outcomes, cognitive function, neuropsychiatric symptoms, and happiness levels of people with dementia. Caregivers’ outcomes included positive aspects of caregiving, perceived stress levels, depressive symptoms, caregiver distress, and happiness levels. Semistructured focus group interviews were conducted with the caregivers to further explore their horticultural experience.

**Results:**

Intervention feasibility was established with a completion rate of 83.78% and an attrition rate of 2.63% (n=1). Significant improvements were detected in caregiver distress (*P*<.05) and the happiness level of people with dementia (*P*<.01). The qualitative findings indicated that HT improved the psychological well-being of both people with dementia and caregivers, enhanced the relationships between caregivers and people with dementia, expanded the caregivers’ social networks, and enhanced the autobiographical memory of people with dementia.

**Conclusions:**

This pilot study provides evidence on the feasibility of using a hydroponic indoor grower to conduct home-based HT for people with dementia and their caregivers. The findings suggest positive effects on the psychological well-being of both people with dementia and their caregivers. Caregivers reported potential positive effects of HT on the autobiographical memory retrieval of people with dementia. Due to the pilot nature of this study, a control group was not employed. Therefore, large-scale randomized controlled trials are encouraged to further confirm the effectiveness of the intervention.

**Trial Registration:**

ClinicalTrials.gov NCT05577975; https://clinicaltrials.gov/study/NCT05577975

## Introduction

With the global aging population, dementia has become a significant public health concern. The World Health Organization estimates that the number of people with dementia worldwide is approximately 55 million, with this figure expected to rise to about 78 million by 2030 and 139 million by 2050 [1]. The global cost of dementia saw a substantial increase of 62% between 2010 and 2019, with the economic burden projected to reach an estimated US $1313.4 billion in 2019. Notably, informal care significantly contributes to roughly half of the total global costs [2]. People with dementia often exhibit behavioral and psychological symptoms of dementia (BPSD), including agitation, anxiety, irritability, depression, apathy, disinhibition, and changes in sleep or appetite [3]. More than 80% of people with dementia were affected by aggression, apathy, agitation, and depression, while around 30% experienced hallucinations and delusions [3,4]. Conceivably, BPSD adversely impacts the capacity to perform activities of daily living (ADL) and the maintenance of interpersonal relationships. This in turn results in significant stress and negative emotions for their family caregivers. For instance, caring for people with dementia has been associated with elevated levels of psychological distress and stress, as well as lower levels of self-efficacy, subjective well-being, and physical health [5]. Due to the significant level of psychological distress and stress involved in caregiving activities, approximately 50% of caregivers of people with dementia are susceptible to major depressive disorder [6]. A high level of caregiver stress is a risk factor for unhealthy behaviors, such as a sedentary lifestyle, inadequate dietary habits, and substance abuse, leading to an increased vulnerability to chronic illnesses among caregivers [7,8]. Caregiver burden is also linked to an increased risk of engaging in abusive behavior towards the care recipient [9]. It is therefore important to develop psychosocial interventions to promote the psychological well-being of both caregivers and people with dementia, as well as to alleviate the BPSD experienced by people with dementia.

Horticultural therapy (HT) has been proposed to be an effective intervention for improving the psychosocial well-being of people with dementia and their caregivers. HT is grounded in the Biophilia Hypothesis, which emphasizes the healing effects of human-plant interactions on physical, mental, and emotional domains [10]. According to the Attention Restoration Theory (ART), engaging with natural environments can restore cognitive fatigue by automatically capturing attention, thereby allowing the replenishment of directed attention capacity [11]. The Stress Recovery Theory (SRT) also proposes that interacting with nature can trigger physiological and psychological stress reduction and elicit positive affect, which in turn facilitates the replenishment of depleted psychological resources after stressful events [12,13]. For instance, HT establishes a sense of control, empowerment, and cooperation, which in turn can facilitate emotional stability when under a state of cognitive overload [14]. The physiological effects of HT on stress reduction in older adults are characterized by lower cortisol, heart rate, and blood pressure [15,16]. It has also been found that increased high α and low α wave activities after a single treatment [17]. Increasing α and theta activity in the brain’s occipital lobes helped decrease anxiety and improve functioning in those with generalized anxiety disorder [18]. In addition, the social interactive nature of HT can facilitate social support for vulnerable groups [19].

More recently, HT has been extended in its application to people with dementia and demonstrated positive effects on BPSD, as well as enhancing physical and cognitive abilities, including memory and orientation, among people with dementia [20]. In the context of caregiving for people with dementia, HT can foster meaningful contact and social interaction between people with dementia and their caregivers [21,22]. A randomized controlled trial (RCT) demonstrated that HT can effectively reduce caregiving burden and promote the quality of life among caregivers of people with dementia [23]. Another RCT reported evidence of the protective effects of HT on the immune system and cognitive functioning, as well as its positive effects on reducing anxiety and promoting social interactions among older adults [24]. Several systematic reviews and meta-analyses have shown reliable effects of HT in improving cognitive functioning in people with dementia [25,26]. For instance, an improved capacity for autobiographical memory retrieval is often observed through engaging in horticultural activities [27]. In addition, HT also demonstrated benefits in alleviating BPSD in people with dementia, as suggested in multiple systematic reviews and meta-analyses [25,27-29].

Traditionally, HT is led by a registered therapist to achieve tailored rehabilitative or vocational goals. HT commonly includes participatory tasks such as cultivating, pruning, weeding, and planting flowers. Ornamental HT may also involve garden tours and nature viewing [16,22]. However, in densely populated areas, HT may not have the luxury of spaces to engage in home-based horticultural activities. The extreme climate in certain areas may create additional difficulties in engaging in outdoor horticultural activities. In addition, for caregivers who may be occupied with caregiving tasks, horticultural activities that require a significant amount of skills and time, such as monitoring the condition of various plants, may pose additional challenges for them [30].

While HT is promising in managing BPSD in people with dementia and improving the quality of life in both people with dementia and their caregivers, it is important to consider the environmental and spatial conditions in which HT is implemented, as these factors may inadvertently impact its effectiveness. In this study, we proposed a novel approach to take advantage of technology to overcome the above limitations and to make horticultural activities more accessible for people with dementia and their caregivers, especially in densely populated areas. In recent years, there has been a growing body of research on the application of technology-assisted HT, such as using virtual reality to deliver HT (eg, VR Garden) [31]. However, there are certain limitations. One of the main drawbacks is the lack of real-world sensory experiences, such as smells and tactile sensations, which are inherent to traditional HT. Additionally, the use of virtual reality may lead to cybersickness, a condition characterized by symptoms similar to motion sickness, which can negatively impact the user experience and therapeutic outcomes [32]. To address these limitations, a hydroponic indoor grower can create a controllable, optimized environment for engaging in horticultural activities in a home-based HT for people with dementia and their caregivers. The hydroponic indoor grower also allows mobile app connectivity to track the progress of plant growth, which can guide people with dementia and their caregivers to engage in horticultural activities. Therefore, this pilot study aimed to address the following research objectives:

1. To examine the feasibility and acceptability of conducting a technology-enhanced home-based HT among people with dementia and their caregivers.
2. To investigate the preliminary effects of the intervention in (1) improving cognitive function and BPSD of people with dementia, (2) promoting positive caregiving experiences in family caregivers, and (3) reducing the caregivers’ level of stress and depressive symptoms.

## Methods

### Study Design

To examine the feasibility and preliminary effects of the technology-enhanced home-based HT on improving the psychological well-being of people with dementia and their caregivers, we used an explanatory sequential multimethods approach. A single-group pre-post design is used for our quantitative assessment. The entire intervention lasted for 11 weeks, with outcome measures taken at baseline (T0) and post intervention (T1). A qualitative approach was used to explore the participants’ experiences in-depth in the HT. Semistructured focus group interviews were conducted with participants after the intervention, with the aim of identifying the strengths, limitations, and difficulties experienced during the program.

### Participants

Participants were recruited from a convenience sample of people with dementia and their caregivers in collaboration with 4 community centers operated by the nongovernmental organization (NGO) that provide gerontology services. Promotional posters were used to advertise the program in NGOs. The program allowed interested participants to enroll independently, while NGO staff reached out to interested individuals to provide further information and assistance. The Hong Kong version of the Montreal Cognitive Assessment-5-Minute (MoCA-5-min) [33] was administered by a research assistant in screening for stages of dementia in potential participants. Eligible participants were then enrolled based on the following inclusion and exclusion criteria. The inclusion criteria for people with dementia were as follows: (1) 65 years or above, (2) diagnosed with any type of dementia at the early to moderate stage, and (3) community-dwelling and living with family caregivers (ie, noninstitutionalized). The criteria for caregivers included the following: (1) at least 18 years old; (2) blood or by-marriage relatives (eg, spouses, siblings, children, and grandchildren) of a person who has been clinically diagnosed with dementia, regardless of its type; and (3) take up caring responsibilities ranging from physical aids (eg, transportation, financial assistance, personal hygiene, and decision-making) to emotional support, and provided most of the daily care and support for the people with dementia (daily contact of at least 4 hours). Participants were deemed ineligible if they had a current medical diagnosis with any acute mental illnesses that would otherwise prevent them from engaging in HT.

An a priori power analysis was performed using G*Power 3.1 [34], based on effect sizes in literature related to the effects of HT on psychological outcomes. A meta-analysis reported an effect size of 0.55 [35], the power analysis was formulated using a 1-tailed *t* test to detect a medium effect size with a power of 0.80 at a *P* value of .05, which resulted in a target sample size of 23. Considering an attrition rate of approximately 15% [36], it was necessary to include more than 27 dyads in the study.

### Ethics Approval

Ethical approval for the study was obtained from the Institutional Review Board of The Hong Kong Polytechnic University (approval number HSEARS20220801002), and the trial was registered in ClinicalTrials.gov (NCT05577975). Prior to participation, all participants (both people with dementia and their caregivers) were required to provide informed consent. The process of obtaining informed consent from people with dementia adhered to the guidelines outlined by the Alzheimer’s Society. A demonstration session was set up to explain the study’s purpose, procedures, risks, benefits, and voluntary nature clearly to both people with dementia and their caregivers. During this session, the research assistant explained the consent form, demonstrated the equipment, and provided an overview of the participation process. People with dementia and their caregivers were allowed to try out some parts of the HT before signing the consent form. Only those people with dementia who were still willing to participate after this experience were allowed to sign the consent form. Ongoing assent was also obtained from participants with dementia throughout the study, and their willingness to participate was regularly monitored and respected.

### Intervention

In the technology-enhanced home-based HT, the activities were also designed based on the Biophilia Hypothesis, with an emphasis on human-plant interactions. We further incorporated interactive components between people with dementia and their caregivers, in which people with dementia participated in the planting process alongside their caregivers. The caregivers assumed responsibility for decision-making and progress monitoring for the planting process, while people with dementia followed directions from caregivers and provided assistance throughout the process. Examples of horticultural tasks performed by caregivers included formulating planting plans, adjusting optimal light conditions, strategizing fruit harvesting schedules, and devising culinary techniques for the harvest. Meanwhile, people with dementia executed tasks such as observation, flower watering, active participation in planting light regulation, and assisting caregivers with harvesting. According to the SRT and ART, these activities are posited to have cognitive and stress restoration effects. Based on the Sensory Integration Theory, these activities can mobilize the 5 senses through multisensory stimulation, tactile input, and olfactory and gustatory experiences [37,38], which can further enhance the cognitive functioning of people with dementia. Furthermore, the interactive components may enhance the dyadic relationship between people with dementia and their caregivers.

**Figure 1:**
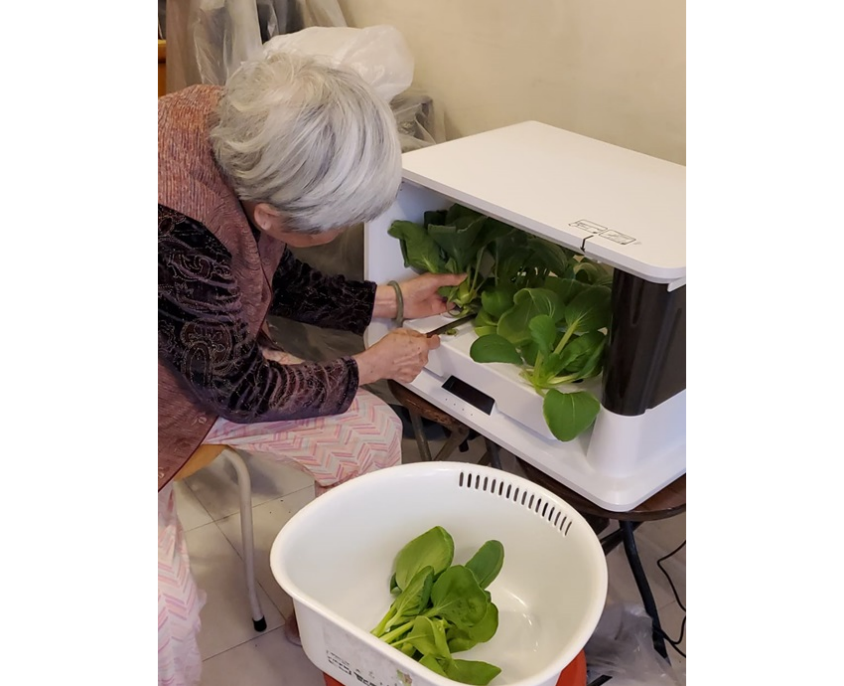
Hydroponic indoor smart grower.

The technology-enhanced home-based HT was facilitated by a hydroponic indoor smart grower, which is a smart device that provides a greenhouse environment with adjustable LED grow lights and smart sensors (Figure 1). Features of the device include automatic watering and a removable water reservoir, as well as built-in smart sensors to detect nutrients, air temperature, humidity, light intensity, water temperature, and water level. The mobile app connectivity via Wi-Fi allows participants to monitor the growth conditions with notifications when water and nutrients need to be replenished, minimizing the probability of plant growing failure. The app can also customize planting plans for each type of plant and automatically adjust to suitable light intensity, resulting in faster plant growth and harvest. Additionally, the app can track the progress of plant growth and guide users to conduct horticultural activities.

Upon the project’s commencement, the hydroponic indoor smart grower was delivered to the enrolled participants. With reference to the optimal therapeutic dose between 100 and 500 minutes of HT for stress reduction [39], the intervention consisted of 3 weekly face-to-face (F-T-F) training sessions, each lasting 90 minutes. This was followed by 8 weeks of home-based horticultural activities (Table 1), with a total weekly time commitment of 1 hour. The program activities are presented in Table 2. A biweekly telephone call schedule was established to follow up on progress and program adherence. Additionally, a social media platform group was created for the participants to ask questions and share their horticultural experiences. The social media platform provided additional insight into participants’ adherence to home-based horticultural activities.

**Table 1:** Intervention schedule of the technology-enhanced home-based horticultural therapy.

	F-T-F^a^ sessions				Home-based horticultural activities with biweekly telephone follow-up							
	Week 1	Week 2	Week 3	Week 4		Week 5	Week 6	Week 7	Week 8	Week 9	Week 10	Week 11
Duration (minutes)	90	90	90	60		60	60	60	60	60	60	60

^a^F-T-F: face-to-face.

**Table 2:** Program content of the technology-enhanced home-based horticultural therapy.

Week	Theme	Objectives	Reality orientation contents
4	Vision; Hearing; Smell	Stimulating the senses of vision, hearing, and smell; focusing on the appearance of the smart grower; encouraging people with dementia-caregiver interaction.	Focusing on LED^a^ light differences according to certain modes of the smart grower; focusing on the water sound when the smart grower performs auto-watering system; smelling the seeds to see if different seeds have different smells; and sharing their sensory information to each other.
5	Smell; Touch; Motor	Stimulating the senses of smell and touch; enhancing visual-spatial skills and executive; promoting motor skills.	Smelling the leaves at the beginning stage of the plants; touching the plants (To see the texture, eg, hard/soft/smooth/rough/solid/fluid); trimming plants; taking photos to record plant growth.
6	Vision; Touch	Stimulating the senses of vision and touch; enhancing visual-spatial skills and executive functioning; enhancing cognitive ability through comparison.	Examining the pattern, shape, size, or height of the vegetables (eg, bok choy vs tomato) in day and night; touching different types of vegetables to see if the textures are different; sharing their sensory information to each other; taking photos to record plant growth.
7	Smell; Vision; Motor; Memory	Stimulating the senses of smell and touch; promoting motor functioning; enhancing visual-spatial skills and executive; enhancing cognitive functioning (memory) through comparison; encouraging people with dementia-caregiver interaction.	Comparing the shape, size, and height of different vegetables to see the differences in the growing process; smelling the leaves or stem of vegetables; pruning the vegetables; sharing and discussing what are the differences from the beginning; taking photos to record plant growth.
8	Motor; Touch; Decision making	Stimulating the senses of touch; promoting motor functioning; stimulating cognitive functioning through decision-making process; enhancing visual-spatial skills and executive.	Touching the leaves or stem of the plants or vegetables to see if the textures are different from the beginning to mid-late stage of planting; planning and discussing which plants to cut; taking photos to record plant growth.
9	Vision; Motor; Decision making	Stimulating the senses of vision; promoting motor functioning; stimulating cognitive functioning through decision-making process; enhancing visual-spatial skills and executive.	Counting the number of vegetables (eg, how many bak choy/tomato?); deciding which vegetables to cut; cutting suitable vegetables; taking photos to record plant growth.
10	Smell; Vision; Motor; Memory	Stimulating the senses of smell and touch; promoting motor functioning; enhancing visual-spatial skills and executive; enhancing cognitive functioning (memory) through comparison; encouraging people with dementia-caregiver interaction.	Comparing the shape, size, and height of different vegetables to see the differences in the growing process; smelling the leaves or stem of vegetables; pruning the vegetables; sharing and discussing what are the differences from the beginning; taking photos to record plant growth.
11	Motor; Vision; Decision-making	Stimulating the senses of vision; promoting motor functioning; stimulating cognitive functioning through decision-making process.	Deciding what type of dishes to cook (salads or pasta); cooking the decided dishes based on the vegetable; recording the odor and color of the dishes; taking photos to record plant growth.

^a^LED: light-emitting diode.

### Measures

#### Overview

Measurements were taken at T0 and T1. The feasibility of the intervention was evaluated by means of attrition rate, attendance rate, and completion rate. The outcome measures for people with dementia included self-care abilities, cognitive function, behavioral symptoms, and happiness levels. Caregivers’ outcomes consisted of positive caregiving experiences, perceived stress levels, depressive symptomatology, caregiver distress, and happiness levels.

#### People With Dementia Outcomes

The Barthel Index was used to measure the capacity to perform ADLs. Barthel Index comprised 10 ADLs, including feeding, bathing, grooming, dressing, using the toilet, transferring (moving from the bed to the chair and back), mobility (on level surfaces), climbing stairs, and controlling bowel and bladder functions [40]. Items are rated based on whether individuals can perform activities independently, with some assistance, or are dependent (scored as 10, 5, or 0). The index yields a total score out of 100, with higher scores indicating greater levels of functional independence [40]. We categorized the Barthel Index scores as follows: 0-20 (total dependence), 21-60 (severe dependence), 61-90 (moderate dependence), 91-99 (mild dependence), and 100 (total independence) [41]. The Barthel Index had excellent test-retest reliability (intraclass correlation coefficient [ICC]≥0.962) [42].

The MoCA-5-min was used to measure the cognitive functions of people with dementia over the telephone, administered by social workers [33]. It comprises 4 domains: attention (immediate recall of 5 words), executive function/language (1-minute verbal fluency), orientation (6-item date and geographic orientation), and memory (delayed recall and recognition of 5 words learned in item 1). The total scores of the MoCA-5-min range from 0 to 30, with higher scores representing a higher level of cognitive function. The MoCA-5-min had an excellent test-retest reliability (ICC=0.89) [33].

BPSD of people with dementia was measured using the Neuropsychiatric Inventory Questionnaire (NPI-Q) [43]. The NPI-Q consisted of 12 neuropsychiatric symptoms, including delusions, hallucinations, agitation/aggression, irritability/depression, anxiety, euphoria/exuberance, apathy/indifference, disinhibition, irritability/instability, abnormal motor behavior, nocturnal behavioral disorders, and appetite/eating disorders. Each symptom is evaluated based on frequency (1=rarely to 4=very often, once or more per day) and severity (1=mild [causing little distress in the patient] to 3=severe [very disturbing to the patient and challenging to redirect]). A total score for each domain is calculated by multiplying the frequency and severity ratings. The overall NPI score is derived by summing the scores across all domains, with a higher score indicating more severe neuropsychiatric symptoms. The NPI-Q has demonstrated satisfactory psychometric properties [43].

The Visual Analogue Scale of Happiness was used to measure the happiness level of people with dementia [44]. The Visual Analogue Scale of Happiness is an analog scale containing 6 faces expressing emotions ranging from great happiness to deep sadness, and people with dementia need to identify which face best represents their mood. Subjects that chose the faces “Little Unhappy,” “Unhappy,” or “Very Unhappy” were prone to exhibit depressive symptoms [45]. The reliability and validity of the scale have been validated [46].

#### Caregivers Outcomes

The Positive Aspects of Caregiving Scale was used to measure caregivers’ positive caregiving experience [47]. It consists of 11 items with 2 subscales: enriching life and affirming self. Participants answered on a 5-point scale ranging from 0 (strongly disagree) to 4 (strongly agree). Scores range from 0 to 44, with higher scores indicating more positive self-perceptions of caregiving. The Positive Aspects of Caregiving Scale demonstrates high levels of internal consistency, with a Cronbach α of 0.89 among the family caregivers of people with dementia in Hong Kong [47].

The Chinese version of the Perceived Stress Scale was used to measure the stress level in caregivers [48]. It is a 10-item measurement rated on a 5-point Likert-type scale, ranging from 0 (never) to 4 (very often). The total score ranges from 0 to 40, with higher scores indicating higher levels of perceived stress. The Perceived Stress Scale showed acceptable levels of psychometric properties, which included an internal consistency reliability of Cronbach α of 0.85 and a test-retest reliability coefficient of 0.85 [49].

The Center for Epidemiological Studies Depression Scale was used to measure depressive symptoms in caregivers over a 1-week recall period [50]. It consists of 20 items and is rated on a 4-point Likert scale, ranging from 0 (rarely or none of the time) to 3 (most or almost all the time). Scores range from 0 to 60, with higher scores indicating greater depressive symptoms. The Center for Epidemiological Studies Depression Scale has demonstrated acceptable psychometric properties, including a test-retest reliability of 0.91 and an internal consistency of 0.86 [51].

The Neuropsychiatric Inventory-Distress Scale (NPI-D) was used to measure caregiving distress [52]. It assesses the psychological distress levels caused by neuropsychiatric symptoms, as reported in the NPI-Q, to the caregivers [43]. Participants rated their distress levels according to the severity of symptoms in people with dementia using a 6-point Likert scale, which ranges from 0 (not at all distressing) to 5 (very severely or extremely distressing). The Cronbach *α* coefficient for the Chinese version of the NPI-D was 0.72 [53].

Perceived happiness was measured using the Subjective Happiness Scale (SHS) [54]. The SHS consists of 4 items; 2 of these assess the current state of happiness, including absolute happiness ratings and relative happiness ratings compared with peers. The remaining 2 items measure trait happiness by asking participants to indicate the extent to which they identify with descriptions of both happy and unhappy individuals. The SHS is rated on a 7-point Likert-type scale, ranging from negative to positive. Responses to the 4 items are averaged, yielding a range from 1 to 7. A higher score indicates greater levels of happiness. The Cronbach α for the Chinese version of the SHS was 0.82, and the test-retest reliability was 0.70 [55].

#### Feasibility Outcomes

Intervention feasibility was assessed using the completion rate, attendance rate, and attrition rate. The attrition rate was calculated by dividing the number of dropouts by the total number of enrolled subjects. The attendance rate was determined by averaging the individual attendance rates, which were calculated by dividing the number of F-T-F sessions attended by the total number of F-T-F sessions. The completion rate was defined as the percentage of participants who attended more than 80% of the sessions, which included home-based horticultural activities and totaled more than 600 minutes.

#### Quantitative Analysis

Data analysis was performed using IBM SPSS (version 23). We first explored patterns of sociodemographic characteristics of the participants. Then, paired-sample *t* tests were used to compare the outcomes before and after the intervention. Unless otherwise specified, the statistical analyses performed did not exceed a 2-tailed α level of 0.05.

#### Qualitative Analysis

Given that caregivers of people with dementia experience varying levels of distress and may respond differently to the intervention, we adopted stratified purposive sampling based on this criterion to enhance the representativeness of the data. The caregiver’s level of distress was assessed using NPI-D, with mild distress categorized as 0-13 points (77.78%), moderate distress as 14-26 points (16.67%), and severe distress as >27 points (5.56%) [52]. Participants for the qualitative study were selected through stratified sampling based on these different levels of distress. A total of 4 semistructured focus groups were conducted with 18 participants. A trained research assistant led the focus groups, asking participants to share their experiences using the smart growers, as well as their interactions with people with dementia during the program. A probing question—“What is your experience related to the use of the device in interacting with your relative?”—was used to facilitate the discussions. The focus group interviews were recorded and transcribed with the participants’ consent.

Thematic analysis was used to analyze the transcribed verbatim; the authors read the texts, generated initial codes, and developed potential themes [56]. The process concluded with data saturation, when no new findings were discovered. Emergent themes were discussed and agreed upon by the researchers. The comprehensiveness of the content analysis was ensured through digital recordings and verbatim transcriptions, which were independently completed and then discussed by 2 researchers. The emergent themes, validated through researcher consensus after thorough discussion, exemplify the methodological rigor underlying the study’s credibility.

## Results

### Quantitative Findings

A total of 50 dyads of people with dementia and their family caregivers were invited to participate in the study, and 38 dyads met the selection criteria and joined the study. The attrition rate was 2.63%, with 1 participant dropping out due to behavioral issues after the first F-T-F training session. The attendance rate for F-T-F sessions was 94.64%. A total of 36 out of 38 dyads attended the first session, while 35 out of 37 dyads attended both the second and third sessions. The completion rate was 83.78%, as 31 out of 37 participants achieved a participation rate of 80% or higher (>600 minutes).

Sample characteristics of people with dementia are presented in Table 3. The majority of participants were male (21/38, 55.3%), with a mean age of 81.8 (SD 8.4) years. Regarding the self-care abilities of the participants, only 21.1% (n=8) exhibited no dependence, while the majority displayed mild to moderate dependence.

**Table 3:** Sociodemographic characteristics of people with dementia (N=38).

Variables			Values
Age (in years), mean (SD)			81.8 (8.4)
Age (in years), range			60-102
**Sex** **, n** **(%)**			
	Male	21 (55.3)	
	Female	17 (44.7)	
**Marital status** **, n** **(%)**			
	Married	23 (60.5)	
	Divorced/Widowed	15 (39.5)	
**Self-care abilities** **, n** **(%)**			
	Total independence	8 (21.1)	
	Mild dependence	25 (65.8)	
	Moderate dependence	5 (13.2)	
	Severe dependence	0 (0.0)	
	Total dependence	0 (0.0)	

Demographic characteristics of caregivers are summarized in Table 4. Female caregivers accounted for 73.7%, with a mean age of 58.4 (SD 8.8) years. The average duration of care provided to people with dementia was 4.7 (SD 0.3) hours per day.

**Table 4:** Sociodemographic characteristics of caregivers (N=38).

Variables			Values
Age (in years), mean (SD)			58.4 (8.8)
Age (in years), range			34-76
**Sex, n (%)**			
	Male	10 (26.3)	
	Female	28 (73.7)	
**Marital status, n (%)**			
	Single	2 (5.3)	
	Married	30 (78.9)	
	Divorced/widowed	6 (15.8)	
**Educational level, n (%)**			
	Primary	10 (26.3)	
	Secondary	20 (52.6)	
	College or above	8 (21.1)	
**Employment status, n (%)**			
	Unemployed/retired	20 (52.6)	
	Employed	18 (47.4)	
**Monthly household income, n (%)**			
	Less than 6000 HKD^a^	11 (28.9)	
	6000-9999 HKD	8 (21.1)	
	10,000-14,999 HKD	10 (26.3)	
	15,000-19,999 HKD	7 (18.4)	
	20,000 HKD or above	2 (5.3)	
**Relationship with people with dementia, n (%)**			
	Spouse	20 (52.6)	
	Children/in-laws	18 (47.4)	
**Living arrangement with people with dementia, n (%)**			
	Same household	23 (60.5)	
	Different household	15 (39.5)	
Duration of care provided to people with dementia, in hours/day (hours), mean (SD)			4.7 (0.3)

^a^HKD: Hong Kong Dollar (1 HKD=US $ 0.13).

For people with dementia, there were significant pre-post improvements in cognitive functions and behavioral and BPSD, although the results did not reach statistical significance. Furthermore, we observed a significant improvement in the happiness level of people with dementia from T0 (mean 7.67, SD 1.40) to T1 (mean 8.79, SD 1.02; *P*<.01; Table 5).

**Table 5:** Outcome measures of the technology-enhanced home-based horticultural therapy for people with dementia (N=37, one participant dropped out).

Variables	T0^a^, mean (SD)	T1^b^, mean (SD)	*P* value	95% CI
Happiness	7.67 (1.40)	8.79 (1.02)	<.01	–1.75 to –0.46
Cognitive functions	14.07 (6.58)	14.36 (6.55)	.61	–1.41 to 0.84
BPSD^c^	10.03 (8.26)	7.72 (5.22)	.20	–1.29 to 5.91

^a^T0: preintervention.

^b^T1: postintervention.

^c^BPSD: behavioral and psychological symptoms of dementia.

Regarding caregivers, at a descriptive level, we observed pre-post improvements in perceived stress, depressive symptoms, and happiness, although the differences did not reach a statistically significant level. Specifically, we observed significant improvements in caregiver distress from T0 (mean 12.93, SD 9.40) to T1 (mean 7.83, SD 8.01; *P*<.05; Table 6).

**Table 6:** Outcome measures of the technology-enhanced home-based horticultural therapy for caregivers (N=37, one participant dropped out).

Variables	T0^a^, mean (SD)	T1^b^, mean (SD)	*P* value	95% CI
Stress	25.72 (6.17)	25.66 (6.00)	.96	–2.89 to 3.03
Depressive symptoms	16.79 (10.70)	17.03 (9.73)	.92	–5.28 to 4.80
Positive aspects of caregiving	31.34 (8.23)	31.00 (10.29)	.89	–4.86 to 5.55
Caregiver distress	12.93 (9.40)^c^	7.83 (8.01)^c^	<.05^c^	0.16 to 10.05^c^
Happiness	6.74 (3.93)	7.08 (4.37)	.41	–1.42 to 3.35

^a^T0: preintervention.

^b^T1: postintervention.

^c^Significant effects.

### Qualitative Findings

#### Overview

A total of 18 caregivers joined the focus group. The majority of them were either children (n=12) or spouses (n=6) of the care recipients. Overall, the qualitative findings from the focus interviews can be summarized under the themes of (1) perceived benefits and (2) perceived challenges of the technology-enhanced home-based HT.

#### Perceived Benefits

Under the theme of perceived benefits, we further identified four subthemes: (1) improved emotional well-being, (2) improved relationship with people with dementia, (3) increased social network among caregivers, and (4) reinforced autobiographical memory retrieval and storytelling abilities.

#### Improved Emotional Well-Being

Caregivers positively recalled how they had enjoyed engaging in horticultural activities at home using the smart grower. They shared their excitement at witnessing the growing process of the vegetables. One participant shared that growing vegetables alleviated her stress, as it had become a meaningful goal in her life. Additionally, she appreciated the colorful lighting of the machine, which they found to be a decorative element in their homes.

Seeing the growth of vegetables is like looking after a baby. The first thing I do every day is to take photos of the vegetables… I also feel happy when I see the attractive symbols on the machine, such as the stars and mountain symbols.

P3

The caregivers were pleased that they could enjoy the fruits of their labor. The vegetables they grew, such as carrots and lettuce, were used to create delicious dishes. They felt a sense of pride and satisfaction after receiving compliments from friends and family members who saw photos of their harvest.

Similar perspectives were also evident in people with dementia. They felt happiness and excitement about the growing process, especially during the harvest. As one caregiver shared,

My father was also involved in the growing of lettuce…He looked at the smart grower and watered the plant every day. He felt happy when he saw the harvest in the smart grower…He could feel the nature at home.

P10

Another caregiver noted that while her husband could not actively participate in the growing process, he enjoyed watching it, particularly the colorful lighting on the smart growing machine.

#### Improved Relationship With People With Dementia

Caregivers found that their relationship with people with dementia improved through home-based horticultural activities. As one caregiver explained,

I put the smart grower near the window, and my husband (people with dementia) will help to water the plant…We are taking care of the plant together…It is just like a toy for us.

P4

These activities offered a way for them to collaborate and grow the vegetables together.

#### Increase Social Network Among Caregivers

Caregivers also reported increased interaction with other caregivers through the home horticultural activities. They were able to ask questions about the smart grower and share photos of their harvest in WhatsApp groups. This facilitated the formation of new friendships, as they had a common topic to discuss.

*I uploaded photos to the WhatsApp group and others posted pictures on the group. So, I see how other people's plants are growing and talk to people.(P1) We have set a WhatsApp groups. When I don't understand sometimes, I will ask in the group. The student assistants will help me in the group and other caregivers will also answer and we will discuss together in the group.* [P9]

The increased social interactions were corroborated by our WhatsApp group records. Participants actively shared recipes for their harvests with each other. Some caregivers displayed their stir-fried Chinese cabbage, while others shared recipes for garlic Chinese cabbage and methods for cooking baby turnip with chieh-qua. More noteworthy was how caregivers enthusiastically shared their planting progress and complimented each other’s growing vegetables. These positive interactions led to the formation of new friendships among participants.

#### Reinforced Autobiographical Memory Retrieval and Storytelling Abilities

One caregiver noted that the smart grower offered an opportunity for people with dementia to reminisce about the past. The people with dementia had experience growing crops in their homeland during childhood, and the home horticultural activities revived those childhood memories. This opportunity facilitated the sharing of historical anecdotes and the exercise of cognitive abilities, such as autobiographical memory retrieval.

Dad is interested in gardening, as growing vegetables with this machine reminded him of his country gardening days, and every now and then he would tell us about his previous experiences.

P13

#### Difficulties

While the horticultural activities generally had positive impacts on people with dementia and their caregivers, there were also some perceived challenges. These challenges can be summarized into two subthemes: (1) operational difficulties in using the smart grower, and (2) light disturbance from the smart grower.

#### Operational Difficulties in Using the Smart Grower

Some caregivers found the smart grower not easy to operate due to its sophisticated design featuring many high-tech components. Issues such as Wi-Fi connection problems were encountered during use. For people with dementia with mild to moderate dependence, the complexity of the operations seemed to diminish their patience and motivation, presenting a barrier to their active participation in the entire growth process.

Sometimes, the elder have no caregivers to help them. So, they can't do it by themselves because they don't understand English very well. For example, the word ‘reset’ they can't understand. Also, they would not know what to do if the Wi-Fi disconnected.

P13

Light Disturbance from the Smart Grower. Additionally, other caregivers expressed concerns about the lighting emitted by the smart grower. They reported using covers or towels to minimize light disturbance at night, indicating a need for better light management features in the device.

The machine emits light and is very disruptive to sleep. Sleeping in a very small house is really affected. When sleeping in the living room, the light turns red and is a little scary. It may have some effect on plants, but not sure. The next day it gives off normal light again.

P14

## Discussion

### Principal Findings

This pilot study aimed to investigate the feasibility of technology-enhanced home-based HT in improving the psychological well-being of people with dementia and their caregivers. Overall, our quantitative results suggested that technology-enhanced home-based HT is a feasible dyadic intervention for both people with dementia and their caregivers. The overall attrition rate was low (2.63%), with a satisfactory completion rate (83.78%). These findings indicate that caregivers and people with dementia are receptive to using technology for engaging in horticultural activities. Further qualitative findings revealed that both people with dementia and their caregivers were excited about using the smart growing machine, as it provided a novel experience for them. They also welcomed the interactive horticultural activities and found that their relationship with each other improved as a result.

The use of technology-enhanced HT has progressively become an increasingly popular approach to delivering HT. For example, virtual reality–based HT has recently been demonstrated as a viable and effective approach in promoting positive psychological outcomes in older adults, such as enhancing self-esteem and mastery [57]. Technology-enhanced HT can mitigate limitations posed by factors such as venue and weather conditions, offering a more flexible and convenient therapeutic approach. In contrast to a virtual reality–based HT, our technology-enhanced home-based HT not only exhibits the benefits of using technologies but also allows participants to touch and feel plants in reality, which can be more relatable to the feeling of nurturing life. According to the Biophilic Hypothesis, actual human-plant interactions play a vital role in exerting the therapeutic effects of HT [58].

Our quantitative results suggested a significant reduction in caregiver distress, aligning with previous studies that used a traditional delivery mode of HT. Previous research has demonstrated that horticultural activities can alleviate the caregiving burden [23]. Our qualitative findings further suggested that home-based horticultural activities may simultaneously enable people with dementia to engage in meaningful and socially interactive experiences with their caregivers, potentially improving their relationships and alleviating caregiver distress. Moreover, our findings indicated a significant increase in happiness levels among people with dementia. These findings also corroborated previous studies using traditional HT that reported a positive emotional impact on people with dementia [59]. Our qualitative findings provided further insights that the heightened levels of subjective well-being could be attributed to participants’ enthusiasm for observing plant growth and acquiring a sense of satisfaction through harvesting activities.

Overall, our findings are in line with the ART, in that perceiving plants in their natural environment has the potential to enhance attention restoration and increase persuasive effects [11,60]. Our findings are also in line with SRT, suggesting that HT is associated with positive affect, which in turn facilitates stress recovery [12,61]. Although we did not observe a significant decrease in perceived stress levels, we found an overall improvement in emotional well-being among caregivers. For example, an increase in emotional well-being is inversely correlated with elevated levels of stress and anger [62]. Consistently, HTs can reduce cortisol levels through engagement in horticultural activities, such as self-harvesting processes, and the consumption of harvested fruits and vegetables [15]. The use of social media groups also facilitated social interactions among caregivers and provided a source of support. The mutual encouragement and compliments about each other’s vegetables, along with sharing recipes for their harvest created positive interactions that extended beyond the initial horticultural activities, offering an additional benefit of alleviating caregiving stress [63-65].

Our qualitative findings suggested that some participants reminisced about their past experiences in farming during the activities. Reminiscing about past events requires the recall of autobiographical memory [66,67], and actively externalizing autobiographical memory for later retrieval (ie, reminiscing about past events) can enhance memory performance and afford greater control over memory encoding processes [68]. While previous studies have suggested that engagement in horticultural activities can enhance the cognitive abilities of people with dementia [21,22], we did not detect a significant pre-post improvement in the cognitive functions of people with dementia. It is plausible that because the MoCA is a global measure of cognition, it may partially explain why we did not detect a significant pre-post improvement in MoCA scores, despite an increasing trend descriptively, as people with dementia primarily improved in memory abilities. However, this interpretation should be made cautiously due to our small sample size. Future studies should incorporate a larger-scale RCT to further investigate the cognitive benefits of the technology-enhanced home-based HT.

### Limitations

This study had several limitations. Due to the pilot nature of the research to establish the feasibility of a technology-enhanced home-based HT, a control group was not employed. Therefore, a full-scale RCT is encouraged to provide further confirmatory evidence about the effectiveness of this intervention. Furthermore, there were only 3 F-T-F sessions provided to offer training and support to the participants. This dosage may have been insufficient. In the qualitative findings, caregivers reported having technical difficulties in using the smart grower at home, such as Wi-Fi connection issues. Previous studies have found similar observations that the older population may have a lower level of readiness for home technology [69]. Therefore, the complexity of the device use may be a significant barrier to the engagement of the older population, particularly for people with dementia [70]. Consequently, it is conceivable that the program outcomes could be further enhanced with more F-T-F sessions and better integrated technical support for people with dementia and their caregivers. A more comprehensive training and support system may help address the operational difficulties encountered with the smart grower technology.

### Conclusion

The findings of this pilot study support the feasibility and offer preliminary evidence of using a hydroponic indoor grower to conduct technology-enhanced home-based HT to improve the psychological well-being of people with dementia and their caregivers. There were positive effects on the emotional well-being of people with dementia and a decrease in caregiver distress. The qualitative findings further suggested improved relationships between people with dementia and caregivers. Additionally, there were potential benefits of this intervention in improving the autobiographical memory retrieval capacity of people with dementia, indicating the cognitive benefits of this intervention. Future studies are encouraged to incorporate a larger and more diverse sample to further evaluate the sustainability of the positive psychological benefits of the technology-enhanced home-based HT for people with dementia and their caregivers.

## Abbreviations

ADL: activities of daily living
ART: Attention Restoration Theory
BPSD: behavioral and psychological symptoms of dementia
F-T-F: face-to-face
HT: horticultural therapy
ICC: intraclass correlation coefficient
MoCA-5-min: Montreal Cognitive Assessment-5-Minute
NGO: nongovernmental organization
NPI-D: Neuropsychiatric Inventory-Distress Scale
NPI-Q: Neuropsychiatric Inventory Questionnaire
RCT: randomized controlled trial
SHS: Subjective Happiness Scale
SRT: Stress Recovery Theory

## References

[1] Shin JH (2022). Dementia epidemiology fact sheet 2022. Ann Rehabil Med.

[2] Wimo A, Seeher K, Cataldi R, Cyhlarova E, Dielemann JL, Frisell O, Guerchet M, Jönsson L, Malaha AK, Nichols E, Pedroza P, Prince M, Knapp M, Dua T (2023). The worldwide costs of dementia in 2019. Alzheimers Dement.

[3] Scales K, Zimmerman S, Miller SJ (2018). Evidence-based nonpharmacological practices to address behavioral and psychological symptoms of dementia. Gerontologist.

[4] Holroyd S, Currie L, Wooten GF (2001). Prospective study of hallucinations and delusions in parkinson's disease. J Neurol Neurosurg Psychiatry.

[5] Ballard C, Corbett A, Chitramohan R, Aarsland D (2009). Management of agitation and aggression associated with alzheimer's disease: controversies and possible solutions. Curr Opin Psychiatry.

[6] Huang SS (2022). Depression among caregivers of patients with dementia: associative factors and management approaches. World J Psychiatry.

[7] Hoffman D, Zucker H (2016). A call to preventive action by health care providers and policy makers to support caregivers. Prev Chronic Dis.

[8] Culberson JW, Kopel J, Sehar U, Reddy PH (2023). Urgent needs of caregiving in ageing populations with alzheimer's disease and other chronic conditions: support our loved ones. Ageing Res Rev.

[9] Wang M, Sun H, Zhang J, Ruan J (2019). Prevalence and associated factors of elder abuse in family caregivers of older people with dementia in central China cross-sectional study. Int J Geriatr Psychiatry.

[10] Niklasson J (2007). Horticultural therapy for homeless people. SLU University Library.

[11] Kaplan S (1995). The restorative benefits of nature: toward an integrative framework. J Environ Psychol.

[12] Ulrich RS, Simons RF, Losito BD, Fiorito E, Miles MA, Zelson M (1991). Stress recovery during exposure to natural and urban environments. J Environ Psychol.

[13] Vacher P, Filaire E, Mourot L, Nicolas M (2019). Stress and recovery in sports: effects on heart rate variability, cortisol, and subjective experience. Int J Psychophysiol.

[14] Kenmochi T, Kenmochi A, Hoshiyama M (2019). Effects of horticultural therapy on symptoms and future perspective of patients with schizophrenia in the chronic stage. Journal of Therapeutic Horticulture.

[15] Yun J, Yao W, Meng T, Mu Z (2023). Effects of horticultural therapy on health in the elderly: a review and meta-analysis. Z Gesundh Wiss.

[16] Nicholas SO, Giang AT, Yap PLK (2019). The effectiveness of horticultural therapy on older adults: a systematic review. J Am Med Dir Assoc.

[17] Hassan A, Qibing C, Tao J (2018). Physiological and psychological effects of gardening activity in older adults. Geriatr Gerontol Int.

[18] Dadashi M, Birashk B, Taremian F, Asgarnejad AA, Momtazi S (2015). Basic Clin Neurosci.

[19] Sempik J, Rickhuss C, Beeston A (2014). The effects of social and therapeutic horticulture on aspects of social behaviour. Br J Occup Ther.

[20] Lin Y, Lin R, Liu W, Wu W (2022). Effectiveness of horticultural therapy on physical functioning and psychological health outcomes for older adults: a systematic review and meta-analysis. J Clin Nurs.

[21] Jarrott SE, Kwack HR, Relf D (2002). An observational assessment of a dementia-specific horticultural therapy program. HortTechnology.

[22] Ferrini F (2003). Horticultural therapy and its effect on people's health. Adv Hortic Sci.

[23] Kim YH, Park CS, Bae H, Lim EJ, Kang KH, Lee ES, Jo SH, Huh MR (2020). Horticultural therapy programs enhancing quality of life and reducing depression and burden for caregivers of elderly with dementia. J People Plants Environ.

[24] Ng KST, Sia A, Ng MKW, Tan CTY, Chan HY, Tan CH, Rawtaer I, Feng L, Mahendran R, Larbi A, Kua EH, Ho RCM (2018). Effects of horticultural therapy on asian older adults: a randomized controlled trial. Int J Environ Res Public Health.

[25] Zhao Y, Liu Y, Wang Z (2022). Effectiveness of horticultural therapy in people with dementia: a quantitative systematic review. J Clin Nurs.

[26] Tu HM, Chiu PY (2020). Meta-analysis of controlled trials testing horticultural therapy for the improvement of cognitive function. Sci Rep.

[27] Lu LC, Lan SH, Hsieh YP, Yen YY, Chen JC, Lan SJ (2020). Horticultural therapy in patients with dementia: a systematic review and meta-analysis. Am J Alzheimers Dis Other Demen.

[28] Whear R, Coon JT, Bethel A, Abbott R, Stein K, Garside R (2014). What is the impact of using outdoor spaces such as gardens on the physical and mental well-being of those with dementia? a systematic review of quantitative and qualitative evidence. J Am Med Dir Assoc.

[29] Xu M, Lu S, Liu J, Xu F (2023). Effectiveness of horticultural therapy in aged people with depression: a systematic review and meta-analysis. Front Public Health.

[30] Kamioka H, Tsutani K, Yamada M, Park H, Okuizumi H, Honda T, Okada S, Park S, Kitayuguchi J, Abe T, Handa S, Mutoh Y (2014). Effectiveness of horticultural therapy: a systematic review of randomized controlled trials. Complement Ther Med.

[31] Xu T, Liao D, Peng X, Liang G, Li Y, Zheng X (2012). Design of virtual reality scenes on horticultural therapy treatment.

[32] Rebenitsch L, Owen C (2016). Review on cybersickness in applications and visual displays. Virtual Reality.

[33] Wong A, Nyenhuis D, Black SE, Law LS, Lo ES, Kwan PW, Au L, Chan AY, Wong LK, Nasreddine Z, Mok V (2015). Montreal cognitive assessment 5-minute protocol is a brief, valid, reliable, and feasible cognitive screen for telephone administration. Stroke.

[34] Faul F, Erdfelder E, Buchner A, Lang AG (2009). Statistical power analyses using G*Power 3.1: tests for correlation and regression analyses. Behav Res Methods.

[35] Panțiru I, Ronaldson A, Sima N, Dregan A, Sima R (2024). The impact of gardening on well-being, mental health, and quality of life: an umbrella review and meta-analysis. Syst Rev.

[36] Siu AMH, Kam M, Mok I (2020). Horticultural therapy program for people with mental illness: a mixed-method evaluation. Int J Environ Res Public Health.

[37] Smith Roley S, Mailloux Z, Miller-Kuhaneck H, Glennon TJ (2007). Understanding Ayres' sensory integration. Sacred Heart University.

[38] Yun HS, Yun SY, Choi BJ (2018). Effects of horticultural activities designed to stimulate five senses on the sensory development of children. J People Plants Environ.

[39] Lu S, Liu J, Xu M, Xu F (2023). Horticultural therapy for stress reduction: a systematic review and meta-analysis. Front Psychol.

[40] Mahoney FI, Barthel DW (1965). Functional evaluation: the Barthel index. Md State Med J.

[41] Filipska-Blejder K, Ślusarz R (2022). Characteristics of the barthel scale in terms of neurological care. PNN.

[42] Dos Santos Barros V, Bassi-Dibai D, Guedes CLR, Morais DN, Coutinho SM, de Oliveira Simões G, Mendes LP, da Cunha Leal P, Dibai-Filho AV (2022). Barthel Index is a valid and reliable tool to measure the functional independence of cancer patients in palliative care. BMC Palliat Care.

[43] Kaufer DI, Cummings JL, Ketchel P, Smith V, MacMillan A, Shelley T, Lopez OL, DeKosky ST (2000). Validation of the NPI-Q, a brief clinical form of the neuropsychiatric inventory. J Neuropsychiatry Clin Neurosci.

[44] Matsubayashi K, Kimura S, Iwasaki T, Okumiya K, Hamada T, Fujisawa M, Takeuchi K, Kawamoto A, Ozawa T (1992). [Evaluation of subjective happiness in the elderly using a visual analogue scale of happiness to analyze the effect of life style and neurobehavioral function on subjective happiness]. Nihon Ronen Igakkai Zasshi.

[45] César KG, Brucki SMD, Takada LT, Nascimento LFC, Gomes CMS, Almeida MCS, Oliveira MO, Porto FHG, Senaha MLH, Bahia VS, Silva TBL, Ianof JN, Spíndola L, Schmidt MT, Jorge MS, Vale PHF, Cecchini MA, Cassimiro L, Soares RT, Gonçalves MR, Smid J, Porto CS, Carthery-Goulart MT, Yassuda MS, Mansur LL, Nitrini R (2014). Performance of the visual analogue scale of happiness and of the cornell scale for depression in dementia in the tremembé epidemiological study, Brazil. Dement Neuropsychol.

[46] Matsubayashi K, Kimura S, Iwasaki T, Okumiya S, Hamada T, Fujisawa M, Takeuchi K, Kawamoto A, Ozawa T (1992). [Evaluation of subjective happiness in the elderly using a visual analogue scale of happiness in correlation with depression scale]. Nihon Ronen Igakkai Zasshi.

[47] Lou VW, Lau BH, Cheung KS (2015). Positive aspects of caregiving (PAC): scale validation among Chinese dementia caregivers (CG). Arch Gerontol Geriatr.

[48] Cohen S, Kamarck T, Mermelstein R (1983). Perceived stress scale. APA PsycTests.

[49] Leung DY, Lam TH, Chan SS (2010). Three versions of perceived stress scale: validation in a sample of Chinese cardiac patients who smoke. BMC Public Health.

[50] Chin WY, Choi EP, Chan KT, Wong CK (2015). The psychometric properties of the center for epidemiologic studies depression scale in Chinese primary care patients: factor structure, construct validity, reliability, sensitivity and responsiveness. PLoS One.

[51] Miller WC, Anton HA, Townson AF (2008). Measurement properties of the CESD scale among individuals with spinal cord injury. Spinal Cord.

[52] Kaufer DI, Cummings JL, Christine D, Bray T, Castellon S, Masterman D, MacMillan A, Ketchel P, DeKosky ST (1998). Assessing the impact of neuropsychiatric symptoms in alzheimer's disease: the neuropsychiatric inventory caregiver distress scale. J Am Geriatr Soc.

[53] Wang T, Xiao S, Li X, Wang H, Liu Y, Su N, Fang Y (2012). Reliability and validity of the Chinese version of the neuropsychiatric inventory in mainland China. Int J Geriatr Psychiatry.

[54] Lyubomirsky S, Lepper HS (1999). A measure of subjective happiness: preliminary reliability and construct validation. Soc Indic Res.

[55] Nan H, Ni MY, Lee PH, Tam WW, Lam TH, Leung GM, McDowell I (2014). Psychometric evaluation of the Chinese version of the subjective happiness scale: evidence from the Hong Kong FAMILY cohort. Int J Behav Med.

[56] Braun V, Clarke V (2006). Using thematic analysis in psychology. Qual Res Psychol.

[57] Fan CC, Choy CS, Huang CM, Chih PS, Lee CC, Lin FH, Guo JL (2022). The effects of a combination of 3D virtual reality and hands-on horticultural activities on mastery, achievement motives, self-esteem, isolation and depression: a quasi-experimental study. BMC Geriatr.

[58] Kaplan R (1973). Some psycholog Ical benefits of gardening. Environ Behav.

[59] Gigliotti CM, Jarrott SE, Yorgason J (2004). Harvesting health: effects of three types of horticultural therapy activities for persons with dementia. Dementia.

[60] Ulrich RS, Simons RF, Losito BD, Fiorito E, Miles MA, Zelson M (1991). Stress recovery during exposure to natural and urban environments. J Environ Psychol.

[61] Zuckerman M (1977). Development of a situation-specific trait-state test for the prediction and measurement of affective responses. J Consult Clin Psychol.

[62] Steptoe A, Deaton A, Stone AA (2015). Subjective wellbeing, health, and ageing. Lancet.

[63] Hui Lin H, Mu Chuan L, Wan Lan C (2017). Benefits of horticultural therapy on emotional and perceived stress of family caregivers: a pilot study. Research in Applied Psychology.

[64] Jueng RN, Lin CY, Huang YH (2023). Systematic review on the positive mental health impact of older adults participation in horticultural activities in long term care facilities. Horticulturae.

[65] Houben M, Van Engen V, Kenning G, Brankaert R (2021). Smile: capturing and sharing personal photos to stimulate social relations and support self-identity in dementia.

[66] Lu X, Kelly MO, Risko EF (2020). Offloading information to an external store increases false recall. Cognition.

[67] Janssen SMJ (2020). Introduction to the cognitive abilities account for the reminiscence bump in the temporal distribution of autobiographical memory. Psychol Rep.

[68] Kelly MO, Risko EF (2019). Offloading memory: serial position effects. Psychon Bull Rev.

[69] Liu L, Stroulia E, Nikolaidis I, Miguel-Cruz A, Rios Rincon AR (2016). Smart homes and home health monitoring technologies for older adults: a systematic review. Int J Med Inform.

[70] Cheung DSK, Ho LYW, Chan LCK, Kwok RKH, Lai CKY (2022). A home-based dyadic music-with-movement intervention for people with dementia and caregivers: a hybrid type 2 cluster-randomized effectiveness-implementation design. Clin Interv Aging.

